# Thermal imaging comparison of Signature, Infiniti, and Stellaris phacoemulsification systems

**DOI:** 10.1186/1471-2415-13-53

**Published:** 2013-10-12

**Authors:** Na Kyung Ryoo, Ji-Won Kwon, Won Ryang Wee, Kevin M Miller, Young Keun Han

**Affiliations:** 1 Department of Ophthalmology, Seoul National University College of Medicine, Seoul, South Korea; 2 Department of Ophthalmology, College of Medicine, Kwandong University, Myongji Hospital, Goyang, South Korea; 3 Department of Ophthalmology, David Geffen School of Medicine at UCLA and the Jules Stein Eye Institute, Los Angeles, CA, USA; 4 Department of Ophthalmology, Seoul Metropolitan Government Seoul National University Boramae Medical Center, #41 Boramae-Gil, Dongjak-Gu, Seoul 156-707, South Korea

**Keywords:** Cataract, Thermal damage, Thermal imaging, Torsional phacoemulsification

## Abstract

**Background:**

To compare the heat production of 3 different phacoemulsification machines under strict laboratory test conditions. More specifically, the thermal behavior was analyzed between the torsional modality of the Infiniti system and longitudinal modalities of the Abbot WhiteStar Signature Phacoemulsification system and Bausch and Lomb Stellaris system.

**Methods:**

Experiments were performed under in-vitro conditions in this study.

Three phacoemulsification handpieces (Infiniti, Signature, and Stellaris) were inserted into balanced salt solution-filled silicone test chambers and were imaged side-by-side by using a thermal camera. Incision compression was simulated by suspending 30.66-gram weights from the silicone chambers. The irrigation flow rate was set at 0, 1, 2, 3, 4, and 5 cc/min and the phacoemulsification power on the instrument consoles was set at 40, 60, 80, and 100%. The highest temperatures generated from each handpiece around the point of compression were measured at 0, 10, 30, and 60 seconds.

**Results:**

Under the same displayed phacoemulsification power settings, the peak temperatures measured when using the Infiniti were lower than when using the other two machines, and the Signature was cooler than the Stellaris. At 10 seconds, torsional phacoemulsification with Infiniti at 100% power showed data comparable to that of the Signature at 80% and the Stellaris at 60%. At 30 seconds, the temperature from the Infiniti at 100% power was lower than the Signature at 60% and the Stellaris at 40%.

**Conclusions:**

Torsional phacoemulsification with the Infiniti generates less heat than longitudinal phacoemulsification with the Signature and the Stellaris. Lower operating temperatures indicate lower heat generation within the same fluid volume, which may provide additional thermal protection during cataract surgery.

## Background

Torsional phacoemulsification has several advantages over longitudinal phacoemulsification, including reduced repulsion and increased followability, which improves phacoemulsification efficiency due to less lens material chattering, shorter surgical time, and decreased tip movement at the incision site [1]. Furthermore, in our previous study of heat production in longitudinal versus torsional phacoemulsification, we have shown that torsional mode generated less heat than longitudinal mode under same stroke length and same applied energy as well as same power setting [2]. However, the difference in power setting between torsional mode and longitudinal mode could affect the heat production of phacoemulsification. Other surgical settings, such as the aspiration flow rate, degree of tip occlusion may also affect heat generation.

The purpose of this study was to compare the heat production of longitudinal and torsional phacoemulsification among 3 phacoemulsification platforms and across selected phacoemulsification power and various flow settings.

## Methods

**Figure 1:**
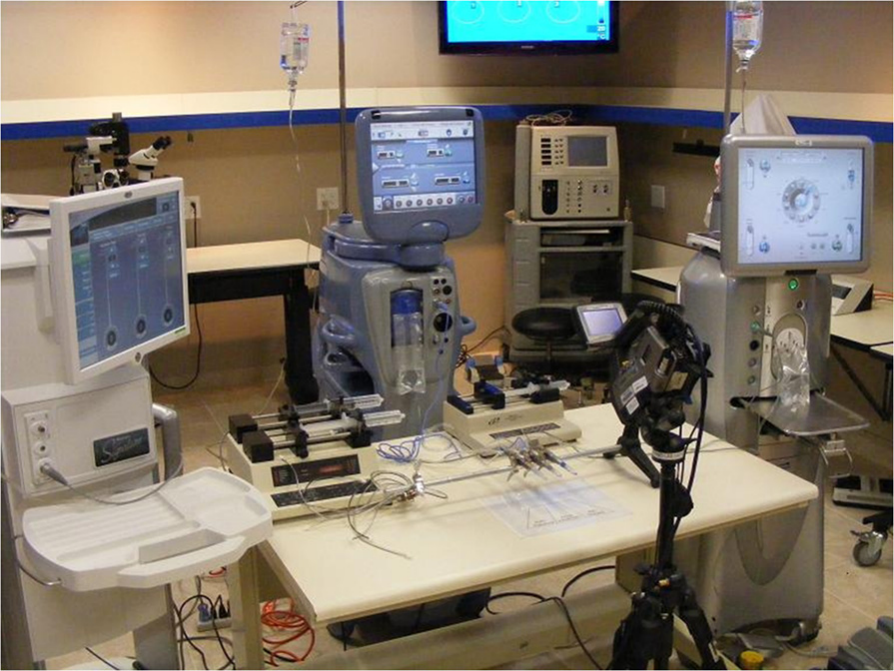
**The basic experiment setup.** The thermal imaging camera, surrounded by 3 phacoemulsification units.

**Figure 2:**
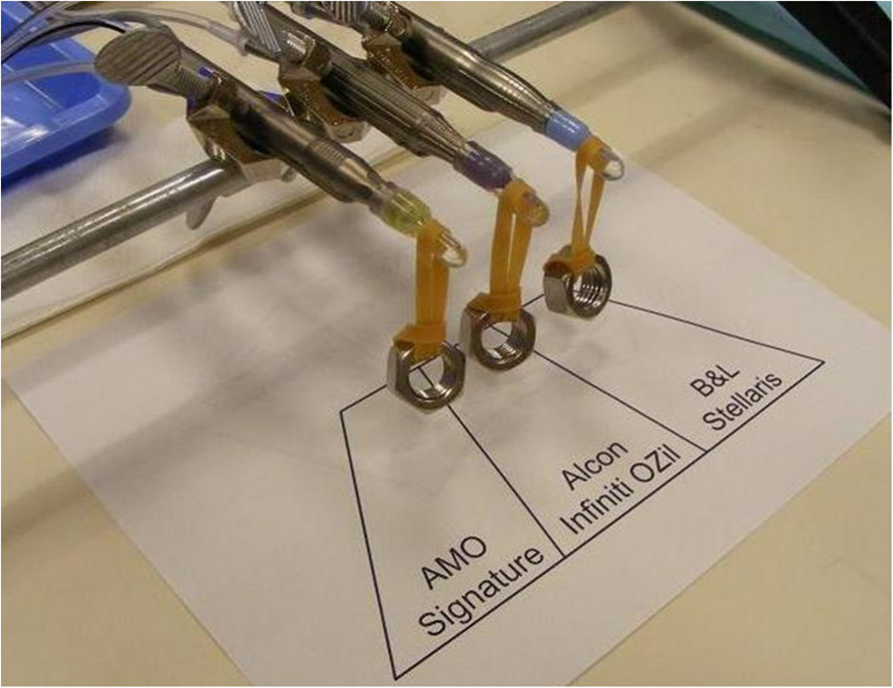
The three probes with 30.66-gm weights in the same plane clamped at a right angle to the thermal camera.

Phacoemulsification systems from 3 major manufacturers (Abbott Medical Optics WhiteStar Signature Phacoemulsification System, Alcon Infiniti Vision System with OZil, and Bausch & Lomb Stellaris Vision Enhancement System) were compared in this experiment (Figure 1). The Intrepid Fluidics Management System, WhiteStar Signature System Fusion Pack, and Stellaris Premium AFS Phaco Pack were each used on their respective machines. The Infiniti used the torsional modality in this study to specifically compare its thermal behavior with the longitudinal modalities available on the other machines. Brand specific phacoemulsification needles – 45-degree Kelman mini-flared ABS tip on an OZil torsional handpiece (Alcon, Inc.), Laminar® Flow straight phaco tip on a WhiteStar Signature longitudinal handpiece (AMO, Inc.), BL3170 straight phaco tip on an Stellaris Vision Enhancement System longitudinal phacohandpiece (Bausch & Lomb, Inc.) – all approximately 0.9 mm in shaft dimension, were used and the distal ends of the brand-matched silicone infusion sleeves were set exactly 1.0 mm from the tips of the needles. The side-ports on the silicone sleeves were oriented at 90 degrees from the beveled ends of the needles. The probes of the 3 phacoemulsification units were each capped with balanced salt solution (BSS)-filled silicone test chambers. The test chambers, able to contain an equal volume of 0.85 cc, were used to imitate in-vivo conditions alike previous studies [2,3]. Elastomeric bands with 30.66-gram weights were suspended from the silicone test chambers to simulate tight corneal incisions (Figure 2). The probes were placed in a common viewing plane perpendicular to a FLIR model P60 ThermaCAM™ (FLIR Systems Inc., North Billerica, Massachusetts) so they could be simultaneously focused upon and imaged side-by-side. The thermal camera captures the infrared radiation that is emitted from an object’s surface and converts it to temperature readings. For our experiments, the FLIR camera was set to display temperatures between 20°C and 90°C (the dynamic range of the camera is −40°C to 120°C). Camera calibration was verified by taking measurements in warm and cold water baths by using the infrared camera and comparing the readings to those from a thermocouple at temperatures across the experimental range.

Phacoemulsification powers were set to 40, 60, 80, and 100%, and aspiration flow rates were set at 0, 1, 2, 3, 4, and 5 ml/min to simulate complete or incomplete occlusion of the tip. To accurately control low aspiration rates through all 3 probes simultaneously, two 60 cc B-D plastic syringes were mounted onto a dual-syringe infusion/withdrawal pump (Cole-Parmer Instrument Co.) and a third 60 cc B-D plastic syringe was mounted onto a Model 33 twin syringe pump (Harvard apparatus). Individual bottles of BSS were placed on a common 4 bar multi-hanger IV-pole.

**Figure 3:**
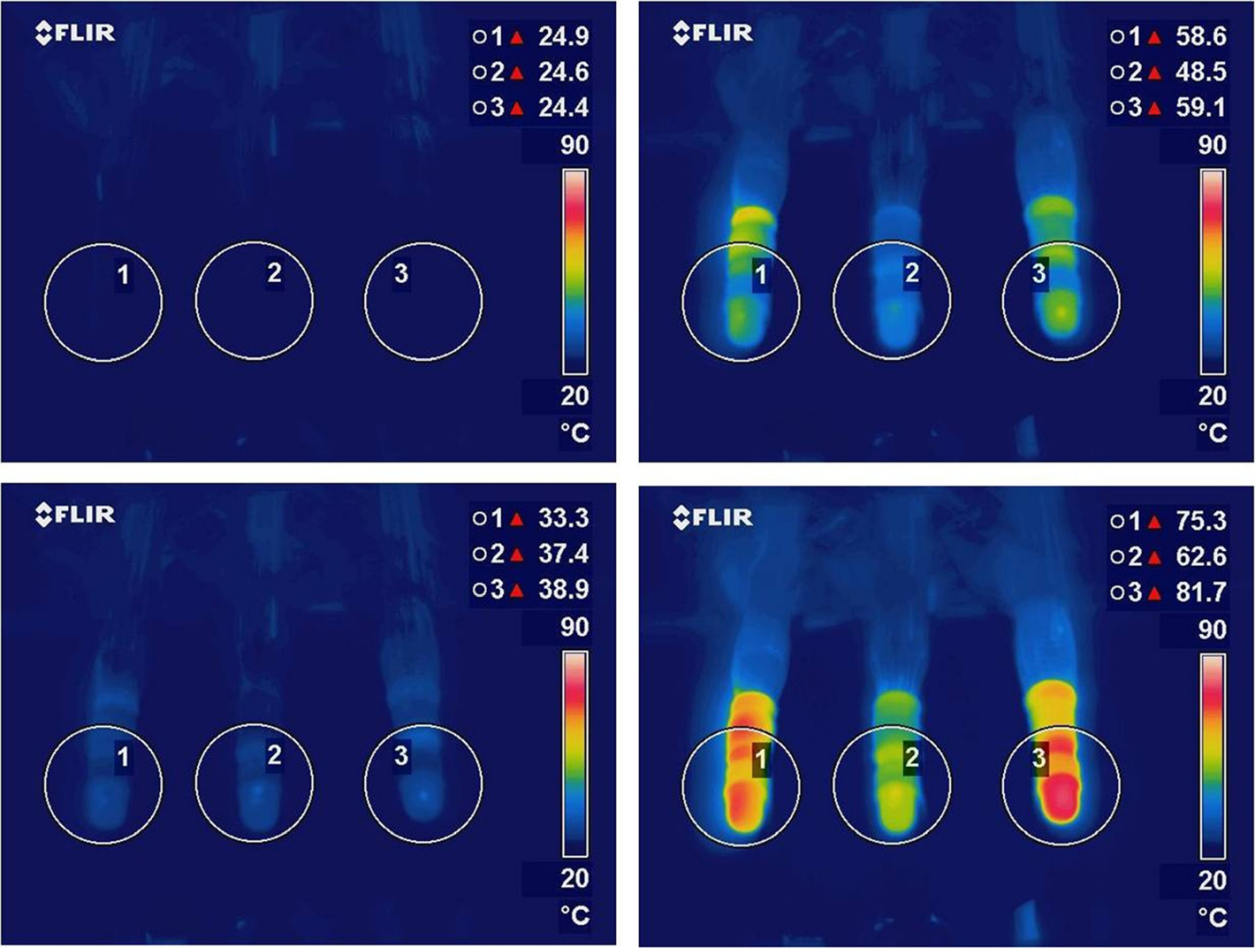
**These thermal images were obtained with 3 handpieces operating at 100% phacoemulsification power with an aspiration flow rate of 0 cc/min.** The upper left image was recorded before power application and subsequent images were recorded 10 (lower left), 30 (upper right), and 60 (lower right) seconds after power application.

The thermal camera captured still images of the probes before power application (at 0 seconds), and then at 10, 30, and 60 seconds after power application. Experiments were run for 1 minute because the volume of fluid in a test chamber takes significantly more time to heat up than a lower volume of aqueous humor in the human eye. The thermal camera has a real-time analysis tool with which a circle can be drawn in a field of interest in order to determine the highest surface temperature inside that circle. We drew circles around each point of contact on each probe where the edge of the elastomeric band pressed against the silicone test chamber. The images were recorded and saved to a compact flash card. Sequential thermal images of silicone test chambers in which the highest temperatures inside the corresponding enumerated circles are displayed in the top right corner are shown in Figure 3.

## Results

**Figure 4:**
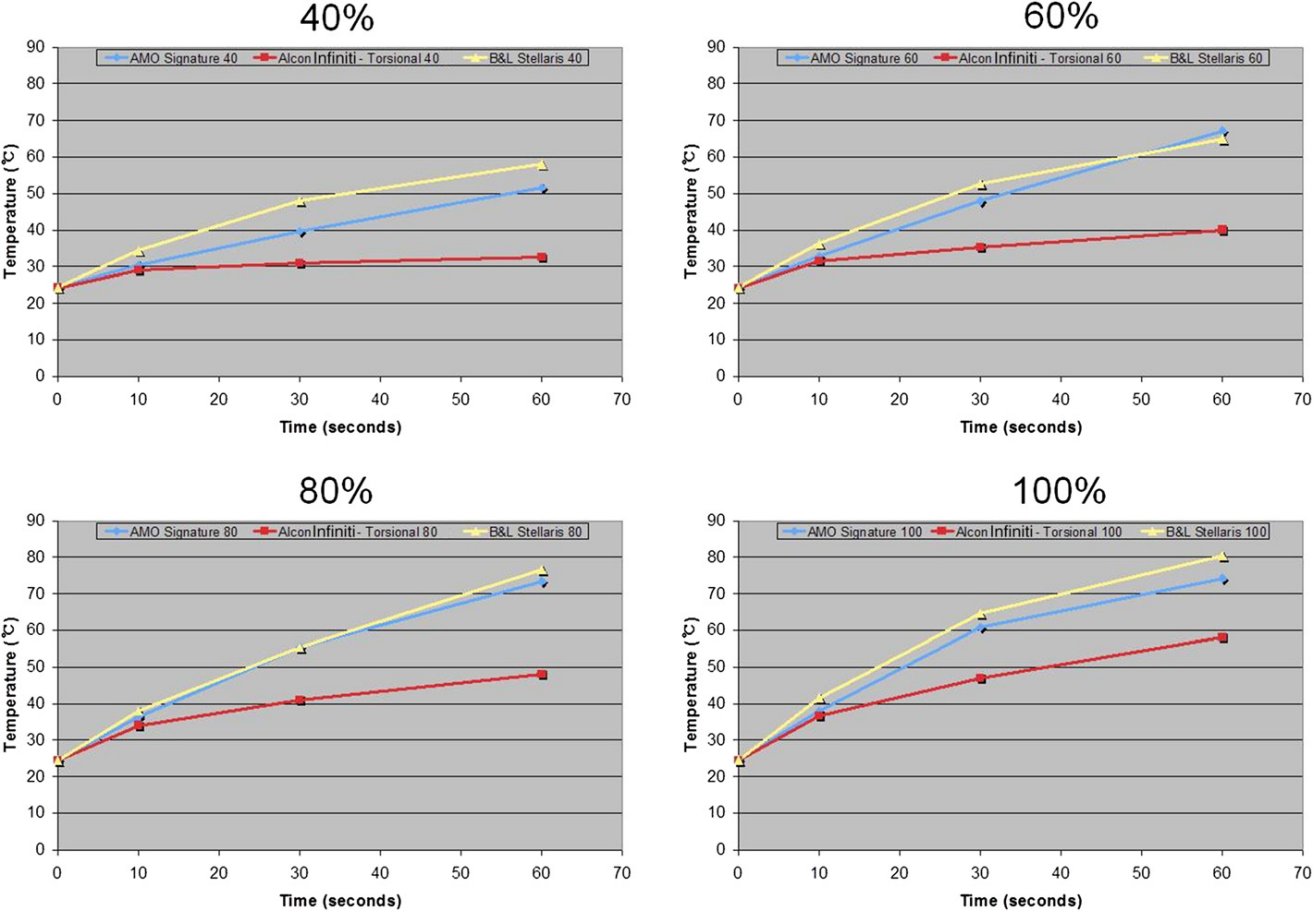
Mean temperature changes with phacoemulsification power settings of 40, 60, 80, and 100% at 0, 10, 30, and 60 seconds after power application in 3 phacoemulsification units.

Mean peak temperature data at 0, 10, 30, and 60 seconds taken at 40, 60, 80, and 100% power settings for each of the 3 machines and modalities are shown in Figure 4. When the 3 machines were compared while using the same displayed power setting, the temperatures measured using Infiniti were lower than the other 2 machines at each time point past 0 seconds. Longitudinal phacoemulsification using the Signature was generally cooler than when using the Stellaris.

**Figure 5:**
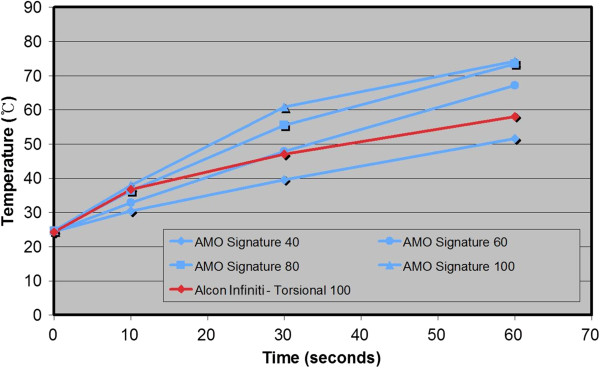
**Within 10 seconds, torsional phacoemulsification with Infiniti at 100% power showed comparable data with the Signature at 80%.** After 30 seconds, the temperature from the Infiniti at 100% power was lower than the Signature at 60%.

**Figure 6:**
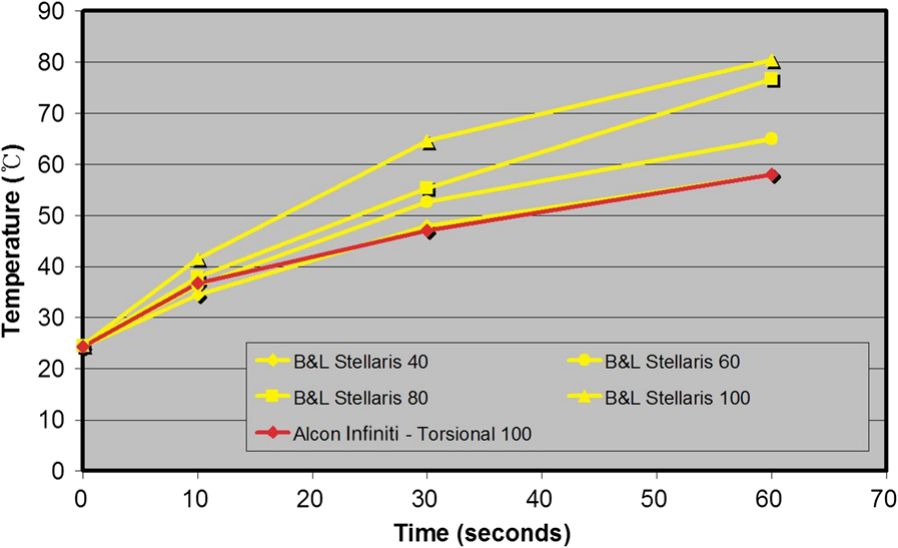
**Within 10 seconds, torsional phacoemulsification with Infiniti at 100% power showed comparable data with the Stellaris at 60%.** After 30 seconds, the temperature from the Infiniti at 100% power was lower than the Stellaris at 40%.

A cross-comparison of torsional phacoemulsification with Infiniti at 100% power at 10 seconds showed temperatures similar to that of the Signature at 80% and the Stellaris at 60%, near 36.5°C. However after 30 seconds, the temperature from the Infiniti at 100% power was closer in value to the temperature from the Signature at 60% and the Stellaris at 40%, at about 47.5°C. In Figures 5 and 6, note that each line that connecting the Infiniti 100 data from 10 to 30 seconds crosses between 2 different lines against the other machines’ data.

For all 3 machines, a higher aspiration flow rate resulted in a cooler tip.

## Discussion

Heat produced during phacoemulsification in cataract surgery, may affect the patients’ cornea, either by direct corneal thermal damage or consequent endothelial cell loss. Therefore, protecting an incision from thermal damage is a prerequisite for patient safety in phacoemulsification cataract surgery. Major contributors of incision temperatures may include incision size, application of ultrasound power, duty cycle, aspiration flow rate, tip design, and the presence of an ophthalmic viscosurgical device (OVD) [4].

Torsional phacoemulsification provides several benefits that help lower the wound temperature. Better followability and reduced repulsion of lens fragments may lead to decreased surgical times and/or potentially lower overall power application [1,5]. Small-angle rotational movement of the shaft also generates less frictional heat, particularly at the wound site. In longitudinal mode, the movement of the tip’s cutting edge is identical to the movement at the incision site along the shaft. In contrast, in torsional mode, the movement of the needle inside the incision site is always less than the stroke of the tip’s cutting edge because the bent tip amplifies the torsional movement and converts it into a nearly horizontal motion [2].

In a previous study we compared the torsional mode and longitudinal mode under the same displayed console power setting, the same stroke length, and the same applied energy on the Infiniti Vision System by using the same experimental approach. We found that torsional phacoemulsification produced less heat than longitudinal phacoemulsification at every experiment [2].

Surgeons can change the parameters in the operative field, and they usually select the lower longitudinal ultrasound power as opposed to torsional mode based on the machine’s display. Recent clinical comparison studies used a maximum power of 60% for conventional ultrasound and a fixed amplitude of 100% for torsional mode [1,6]. Because surgeons have a tendency to use higher displayed power for torsional mode, the use of longitudinal mode with a lower power setting should generate less heat in the operative field. In this study, we attempted to determine which selected power settings were comparable between torsional and longitudinal phacoemulsification modes and among different manufactured systems.

In the present study, thermal data from the 100% torsional mode with the Infiniti was comparable to longitudinal mode with the Signature at 80% power and the Stellaris at 60% power at 10 seconds. At 30 seconds, the temperature from the Infiniti at 100% power was lower than the temperature from the Signature at 60% and the Stellaris at 40%. A more rapid increase in temperature over time indicates that the heat generation rate is also increased. Therefore, this study found that torsional phacoemulsification produces less heat than longitudinal phacoemulsification and suggests that the thermal advantage from torsional mode may be best realized when working on a hard nucleus that would require more time for phacoemulsification.

A potential criticism of this study is that it did not consider the efficiency of torsional mode attributable to reduced repulsion and increased followability. The oscillatory movement of the torsional tip provides steady and constant contact with the nucleus, which results in a decreased phacoemulsification time.

In this study, as mentioned before, we have focused on the torisonal mode of the Infiniti system and longitudinal mode of other systems. Such modality was selected to objectively compare the heat generation in such conditions. However, combination of different modalities and recent technological advances, also provide other options [3,7,8]. In a study with in-vitro testing on human brunescent nuclei, new variations of non-longitudinal ultrasound (i.e., OZil-IP and Ellips-FX) showed to be more efficient than previous OZil and Ellips technology, in certain aspects, such as time efficiency, which may be correlated with heat generation [8]. Further studies under other parameters or with different systems are encouraged.

## Conclusions

This study offers surgical tips from a practical perspective by comparing the true heat generated under diverse power settings in torsional and longitudinal mode with the passage of time. In particular, it confirms that the advantages of torsional phacoemulsification increase in beginners whose phacotime extends 10 seconds.

## Competing interests

The authors declare that they have no competing interests.

## Authors’ contributions

NKR performed all experiments and draft the manuscript. JWK carried out the data collection and analysis. WRW participated in the thermal imaging study setting and helped to draft the manuscript. KMM participated in the design of the study and helped to revised the manuscript. YKH participated in the design of the study and performed experiments. All authors read and approved the final manuscript.

## Authors’ information

NKR is a chief resident in Department of Ophthalmology, Seoul National University Hospital.

JWK is an Associate Professor in Kwandong University College of Medicine.

WRW is a Professor in Seoul National University College of Medicine.

KMM is the Kolokotrones Professor of Clinical Ophthalmology, David Geffen School of Medicine at UCLA.

YKH is an Associate Professor in Seoul National University College of Medicine and Head in the Department of Ophthalmology, Boramae Medical Center.

## Pre-publication history

The pre-publication history for this paper can be accessed here:

http://www.biomedcentral.com/1471-2415/13/53/prepub
